# Epigallocatechin Gallate Alleviates Lipopolysaccharide-Induced Intestinal Inflammation in Wenchang Chicken by Inhibiting the TLR4/MyD88/NF-κB Signaling Pathway

**DOI:** 10.3390/vetsci12030225

**Published:** 2025-03-02

**Authors:** Xin Xu, Kunpeng Li, Qian Liu, Haiwen Zhang, Lianbin Li

**Affiliations:** 1 Key Laboratory of Tropical Animal Breeding and Epidemic Disease Research of Hainan Province, School of Tropical Agriculture and Forestry, Hainan University, Haikou 570100, China; 13545069507@163.com (X.X.); lkpeng2022@163.com (K.L.); a2469478224lq@163.com (Q.L.); 007zhanghaiwen@163.com (H.Z.); 2 School of Life and Health Sciences, Hainan University, Haikou 570228, China

**Keywords:** epigallocatechin gallate, Wenchang chicken, intestinal inflammation, Lipopolysaccharide, TLR4/MyD88/NF-κB

## Abstract

Intestinal inflammation significantly compromises broiler health and adversely affects growth performance. Epigallocatechin gallate (EGCG) was found to maintain the gut health of animals. However, the role and mechanism of EGCG in preventing lipopolysaccharide (LPS)-induced intestinal inflammation in chicks have not yet been fully elucidated. In the 35-day study, 140 one-day-old Wenchang chickens were randomly assigned to four treatments: CON (basal diet), LPS (basal diet + 1 mg/kg body weight (BW) LPS), L-EGCG (basal diet + 40 mg/kg BW EGCG + 1 mg/kg BW LPS), and H-EGCG (basal diet + 60 mg/kg BW EGCG + 1 mg/kg BW LPS). On days 31, 33, and 35 of age, broilers in the LPS, L-EGCG, and H-EGCG treatments received intraperitoneal injections of LPS. The LPS reduced jejunal villus height, villus height/crypt depth ratio, *Claudin1* mRNA, catalase (CAT) activity, and interleukin-10 (IL-10) levels compared to CON while elevating diamine oxidase (DAO), interleukin-1β (IL-1β), and tumor necrosis factor α (TNF-α). EGCG improved growth performance in LPS-challenged broilers, elevating jejunal villus height and *Claudin1/ZO-1* mRNA with reduced serum DAO. It enhanced antioxidant capacity via increased serum total antioxidant capacity (T-AOC), total superoxide dismutase (T-SOD), CAT, glutathione peroxidase (GSH-Px) activities, and a decreased malondialdehyde (MDA) concentration. Concurrently, EGCG lowered IL-1β/TNF-α and raised IL-10 in serum/jejunum. Crucially, EGCG suppressed jejunal *TLR4/MyD88/NF-κB* mRNA and protein expression under LPS. These findings demonstrate EGCG’s protective role against LPS-induced intestinal inflammation in Wenchang chickens through TLR4/MyD88/NF-κB pathway inhibition.

## Simple Summary

Wenchang chicken, an indigenous broiler breed from Hainan Island, China, is prized for its superior meat quality. Due to the tropical climate of Hainan and their extended growth cycle, these chickens exhibit increased susceptibility to intestinal inflammation. Intestinal inflammation significantly compromises broiler health and adversely affects growth performance. Epigallocatechin gallate was found to maintain the gut health of animals. However, the role and mechanism of epigallocatechin gallate in preventing lipopolysaccharide-induced intestinal inflammation in chicks have not yet been fully elucidated. This study hypothesizes that epigallocatechin gallate alleviates lipopolysaccharide-induced intestinal inflammation, thereby improving growth performance in Wenchang chickens. Therefore, we established a lipopolysaccharide-induced intestinal inflammation model in Wenchang chickens to investigate epigallocatechin gallate’s effects on inflammatory response and the underlying mechanisms. Our results demonstrate that epigallocatechin gallate alleviated lipopolysaccharide-induced intestinal inflammation and improved growth performance in Wenchang chickens. Our findings provide new insights into the ability of epigallocatechin gallate to promote intestinal health in Wenchang chickens and suggest that epigallocatechin gallate has the potential to be an additive for improving the intestinal health of Wenchang chicks.

## 1. Introduction

Intestinal inflammation can have a series of adverse reactions on poultry health, as it can cause intestinal damage, leading to impaired intestinal function [1]. Currently, commercial poultry, especially chicks, are highly susceptible to subclinical and clinical intestinal inflammation influenced by inflammatory triggers prevalent in intensive production settings, such as pathogen infections, high temperatures, and other various stressors [2]. When intestinal inflammation occurs in chicks of 2–6 weeks of age, it can result in a total mortality of 10–40% [3]. While dietary antibiotics alleviate intestinal inflammation, the presence of antibiotic residue poses a threat to the health of both animals and humans [4]. Therefore, the effort to reduce antibiotic use in animal production has led to an increased interest in exploring alternative, advantageous, and environmentally friendly dietary supplements.

Several studies have explored natural plant extracts as dietary supplements to enhance immune function and alleviate intestinal inflammation in animals [5]. Among these, epigallocatechin gallate (EGCG) has gained significant attention due to its diverse biological activities, including antioxidant [6] and anti-inflammatory activities [7]. EGCG is a catechin monomer isolated from tea and the most abundant active substance in tea polyphenols [8]. EGCG demonstrated protective properties against inflammation in various cellular and animal models. For instance, in rat cardiomyocytes H9c2 cells, EGCG mitigated the inflammation generated by lipopolysaccharide (LPS) by inhibiting the NF-κB pathway [9]. EGCG supplementation was shown to mitigate intestinal inflammation and strengthen barrier integrity in mice treated with dextran sulfate sodium (DSS) [10]. In broiler production, dietary supplementation of green tea extract (GTE) improved the antioxidant level and immune stress ability of broilers [11]. In addition, studies had demonstrated that supplementing feed with 300 mg/kg and 600 mg/kg EGCG yields multiple benefits for heat-stressed broilers: it improves growth performance [12], mitigates small intestinal structural damage, and enhances antioxidant capacity [13]. However, there are relatively few studies on EGCG in regard to preventing intestinal inflammation in chicks caused by LPS.

The primary cause of intestinal disease is the LPS produced by Gram-negative bacteria [14], and its intraperitoneal injection has been widely used to experimentally induce intestinal injury models in broilers [15]. However, there is insufficient evidence regarding the impact of EGCG modulation on the function of the intestinal tract and the inflammatory reaction in young broilers, especially in the LPS-induced model.

The Wenchang chicken, an indigenous broiler breed from Hainan Island, China, is prized for its superior meat quality. Wenchang chickens have a growth cycle of 120–180 days, with the first 35 days being classified as the chick stage. The growth rate of Wenchang chickens is slower than that of commercial fast-growing broiler breeds, with female broilers attaining a body weight of 1350–1650 grams at 120 days of age. Due to the tropical climate of Hainan and their extended growth cycle, these chickens exhibit increased susceptibility to intestinal inflammation [16]. Therefore, Wenchang chickens urgently need to find natural additives that can improve their intestinal health. This study hypothesizes that EGCG alleviates LPS-induced intestinal inflammation, thereby improving growth performance in Wenchang chickens.

This study established an intestinal inflammation model in Wenchang chickens by intraperitoneal injection of LPS. Within this model, EGCG was orally gavaged to investigate its alleviating effects on intestinal inflammation and explore the underlying molecular mechanisms in chicks. This study will provide experimental evidence supporting the application of EGCG as a potential dietary supplement to improve intestinal health and enhance production performance in Wenchang chickens.

## 2. Materials and Methods

### 2.1. Animals and Experimental Design

A total of 140 one-day-old female Wenchang chicks were acquired from the Hainan (Tanniu) Wenchang Chicken Company in Hainan, China. Female Wenchang chickens are primarily used for fattening and sale in Hainan markets, so female chicks were selected for this study. These chicks were then placed at the Hainan University Animal Laboratory Base, where they were subjected to regular conditions and received consistent management. After a 7-day acclimation period, chicks (76.62 ± 0.71 g, n = 140) were randomly allocated into four treatment groups with five replicates each: CON (basal diet), LPS (basal diet + 1 mg/kg body weight (BW) LPS), L-EGCG (basal diet + 40 mg/kg BW EGCG + 1 mg/kg BW LPS), and H-EGCG (basal diet + 60 mg/kg BW EGCG + 1 mg/kg BW LPS). This study adopted the standard feed formulation commonly used during the chick stage of Wenchang chickens [17]. The composition and nutritional levels of the basal diet for the chicks are shown in Table 1. From days 8 to 35, EGCG groups received a daily oral gavage of EGCG at their respective doses, while CON and LPS groups received equivalent volumes of sterile PBS. On days 31, 33, and 35, LPS, L-EGCG, and H-EGCG groups received intraperitoneal LPS injections (1 mg/kg BW), while the CON group received equivalent saline injections. During the gavage process, we used a syringe to draw the corresponding volume of EGCG solution, restrained the chicks, gently opened their beaks, and injected the EGCG solution into their mouths.

Each replicate was placed separately in a 1 m^2^ cage, with 7 chickens in each cage. The temperature for one-week-old chicks is 33–35 °C. Thereafter, the temperature decreased by 1 °C every week until the fifth week, when room temperature was used, and the lighting time was reduced by 4 h every week until the fourth week, when natural lighting was maintained. Feed and water were provided ad libitum throughout the 28-day experiment.

LPS (CAS: 93572-42-0) was purchased from Beijing Mreda Technology Company (Beijing, China) and was derived from *Escherichia coli* 055:B5 through purification by trichloroacetic acid extraction. EGCG (CAS: 989-51-5) with a purity of 98% was purchased from Xi’an Tongze Biotechnology Co., Ltd. (Xi’an, China). EGCG is extracted from the leaves of green tea, appearing as a white crystalline powder that is soluble in ethanol, dimethylformamide, and water.

### 2.2. Growth Performance

The amount of feed consumed by each treatment was recorded over a period of 35 days. Weight measurements were taken on day 7 and day 35. Data from replicates were used to calculate body weight gain (BWG), average daily feed intake (ADFI), and the feed conversion ratio (FCR).

### 2.3. Sample Collection

On day 35, after a 12 h fasting period, two broilers were randomly selected from each replicate (n = 10 per treatment) and sacrificed by cervical dislocation. Blood samples were collected in coagulation tubes, centrifuged (1500× *g*, 15 min, 4 °C), and the serum was stored at −80 °C. The jejunal segments were excised and divided into two portions: one portion was fixed in 4% paraformaldehyde, and another was rinsed with saline and stored at −80 °C for subsequent mRNA and protein expression analyses.

### 2.4. Intestinal Morphology

The intestine segments were removed from the paraformaldehyde and embedded with paraffin wax; then, the tissue was cut to a thickness of 4 microns and finally stained with Hematoxylin–Eosin (HE). The slices that had been stained were subsequently examined using a microscope. Villus height (VH) was defined as the top to the bottom of the intact villi, and crypt depth (CD) was defined as the top to the bottom of the crypt. Based on these data, the ratio of villus height to crypt depth (VH:CD) was calculated.

### 2.5. Antioxidant Activity

Catalase (CAT) and glutathione peroxidase (GSH-Px) activities, malondialdehyde (MDA) concentration, total superoxide dismutase (T-SOD), and total antioxidant capacity (T-AOC) activities were measured using commercial kits (Nanjing Jiancheng Bioengineering Research Institute, Nanjing, China) following the provided instructions for quantitative analysis. All antioxidant indicators were measured using serum samples from the same batch, but in different groups, and each measurement was performed in triplicate simultaneously.

### 2.6. Detection of Inflammation and Assessment of Barrier Function

Diamine oxidase (DAO) in serum and interleukin-1β (IL-1β), interleukin 10 (IL-10) and tumor necrosis factor α (TNF-α) in serum and jejunum were determined by a chicken- specific ELISA kit (Amo Lun Changshuo Biotechnology Co., Ltd., Xiamen, China) with catalog numbers ED-69214 for DAO, ED-60036 for IL-1β, ED-60031 for IL-10, and ED-60161 for TNF-α. The tissues were rinsed with pre-cooled PBS to remove the residual blood, and the tissues and corresponding PBS were added to the glass homogenizer for grinding at a 1:9 weight to volume ratio; then, the homogenate was centrifuged at 5000 rpm for 10 min to absorb the supernatant for detection. In addition, the protein concentration was detected using the bicinchoninic acid (BCA) kit before, finally, the content of cytokines in each milligram of protein was calculated.

### 2.7. Total RNA Extraction and Real-Time Quantitative PCR (RT-qPCR) Analysis

The jejunum segments were decomposed with Trizol lysate (Tiangen Biochemical Technology Co., Ltd., Beijing, China) for RNA isolation. The extracted RNA was first subjected to purity testing, where the A260/280 ratio was measured using a Microspectrophotometer (Hangzhou Aosheng Instrument Co., Ltd., Hangzhou, China) to ensure it fell within the range of 1.8–2.1, and the A260/230 ratio was between 2.0 and 2.2. The integrity of the RNA was then assessed by observing the 28S and 18S rRNA bands through agarose gel electrophoresis. One microgram of total RNA was converted into complementary DNA (cDNA) using the HiScript First Strand cDNA Synthesis Kit (Takara Biotechnology Co., Ltd., Beijing, China). Primers for a RT-qPCR analysis were specifically formulated using sequences obtained from the GenBank repository (Table 2). The RT-qPCR process consisted of an initial denaturation at 95 °C for 30 s, followed by 40 cycles of denaturation at 95 °C for 5 s and annealing/extension at 65 °C for 30 s. Prior to conducting the formal qPCR, a preliminary experiment was performed on the primers and the reverse-transcribed cDNA. Each sample and gene was set up in triplicate, and all genes showed stable expression. The CT values for the target genes were within the range of 18–20 and did not exceed 35. Target genes were normalized to *β-actin* as an internal control, and relative expression levels were calculated using the 2^−ΔΔCT^ approach.

### 2.8. Western Blot Analysis

Jejunal proteins were extracted using radioimmunoprecipitation assay (RIPA) buffer containing a 1% protease inhibitor cocktail (Beyotime Biotechnology, Shanghai, China). Following centrifugation (12,000× *g*, 10 min), the total protein concentration was determined by BCA assay (Biosharp Biotechnology Co., Ltd., Hefei, China) according to manufacturer’s instructions. All samples were adjusted to 2 μg/μL using RIPA buffer with 5× loading buffer (Beyotime Biotechnology). Proteins were separated by sodium dodecyl sulfate polyacrylamide gel electrophoresis and transferred to polyvinylidene fluoride membranes. Membranes were incubated overnight with primary antibodies against β-actin (66009-1-IG, 1:10,000, Proteintech Group, Inc., Wuhan, China), TLR4 (WL00196, 1:500, Wanlei Bio Co., Ltd., Shenyang, China), MyD88 (WL02494, 1:500, Wanlei Bio Co., Ltd., Shenyang, China), and phospho-p65(p-p65) (bs-0982R, 1:1000, Beijing Biosynthesis Biotechnology Co., Ltd., Beijing, China). Protein expression was normalized to β-actin. The quantification procedure was performed as follows: (1) the internal reference protein and target protein of each sample were quantified using ImageJ (64-bit Java 1.6.0_20); (2) the quantified value of the target protein for each sample was divided by the quantified value of the internal reference protein; (3) the average ratio of the control group was calculated; (4) all ratios were divided by this average value.

### 2.9. Statistical Analysis

Data were analyzed using SPSS software (version 25.0). A one-way analysis of variance (ANOVA) was used to analyze the experimental results, and the least significant difference (LSD) method was used to compare the significance of multiple data. A significance level of *p* < 0.05 was deemed notably significant. Throughout, * *p* < 0.05, ** *p* < 0.01; *** *p* < 0.001, **** *p* < 0.0001, not significant (ns) *p* > 0.05. All graphical representations were created using GraphPad Prism (version 10.1.2).

## 3. Results

### 3.1. Effects of EGCG on the Growth Performance of LPS-Challenged Wenchang Chicks

**Figure 1:**
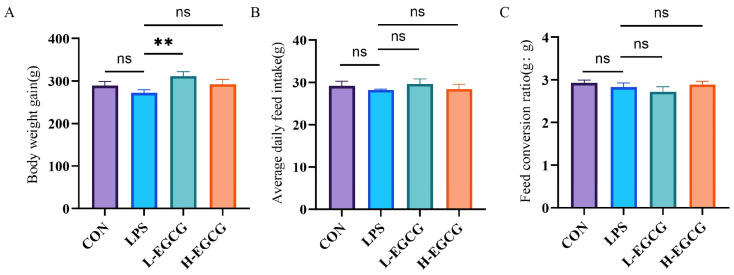
Effects of epigallocatechin gallate (EGCG) on growth performance of Wenchang chicks challenged with lipopolysaccharide (LPS). (**A**) Body weight gain (BWG); (**B**) average daily feed intake (ADFI); (**C**) feed conversion ratio (FCR). The results are expressed as mean ± the standard error of the mean (** *p* < 0.01, not significant (ns) *p* > 0.05).

Compared to the LPS treatment, the BWG in the L-EGCG (40 mg/kg) treatment was higher (*p* < 0.05, Figure 1A) during days 7–35. No mortality occurred during the experiment.

### 3.2. Effects of EGCG on the Jejunum Morphology of LPS-Challenged Wenchang Chicks

**Figure 2:**
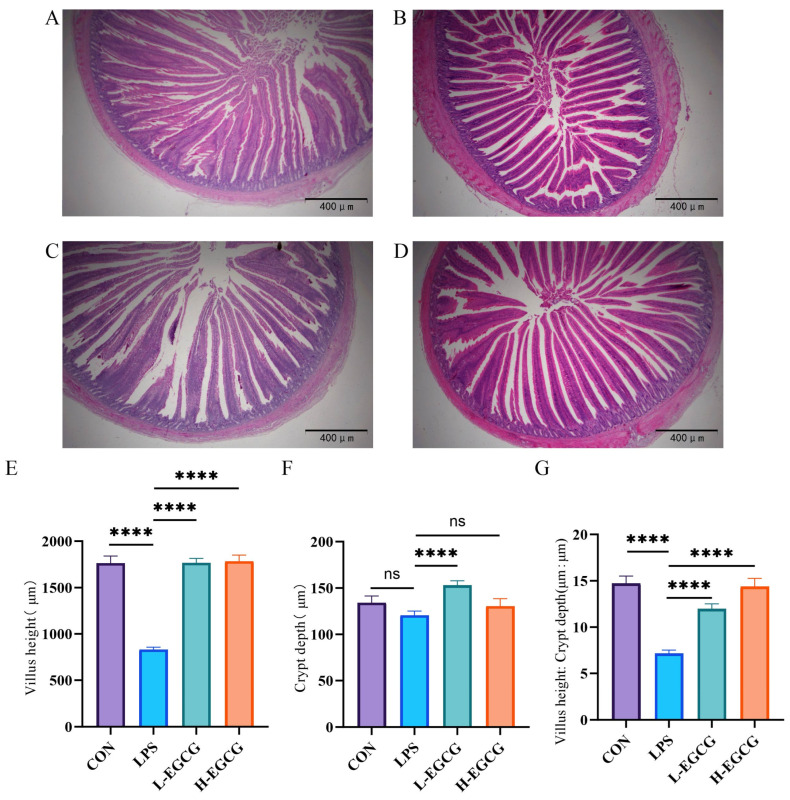
The effects of EGCG on the jejunum morphology of Wenchang chicks challenged with LPS. The morphological structure of the jejunum of Wenchang chicks after (**A**) CON treatment, (**B**) LPS treatment, (**C**) L-EGCG treatment, (**D**) H-EGCG treatment. (**E**) The jejunal villus height, (**F**) jejunal crypt depth, (**G**) jejunal villus height/crypt depth ratio of the jejunum. The results are expressed as mean ± the standard error of the mean (**** *p* < 0.0001, not significant (ns) *p* > 0.05).

LPS injection induced evident structural damage to jejunal villi, characterized by shortened VH and altered morphology (Figure 2A–D). Compared to controls, the jejunal VH and VH:CD in the LPS treatment were lower (*p* < 0.001, Figure 2E,G). Compared to LPS treatment, the VH and VH:CD were higher (*p* < 0.001, Figure 2E,G) after the EGCG gavage process.

### 3.3. Effects of EGCG on the Jejunum Permeability of LPS-Challenged Wenchang Chicks

**Figure 3:**
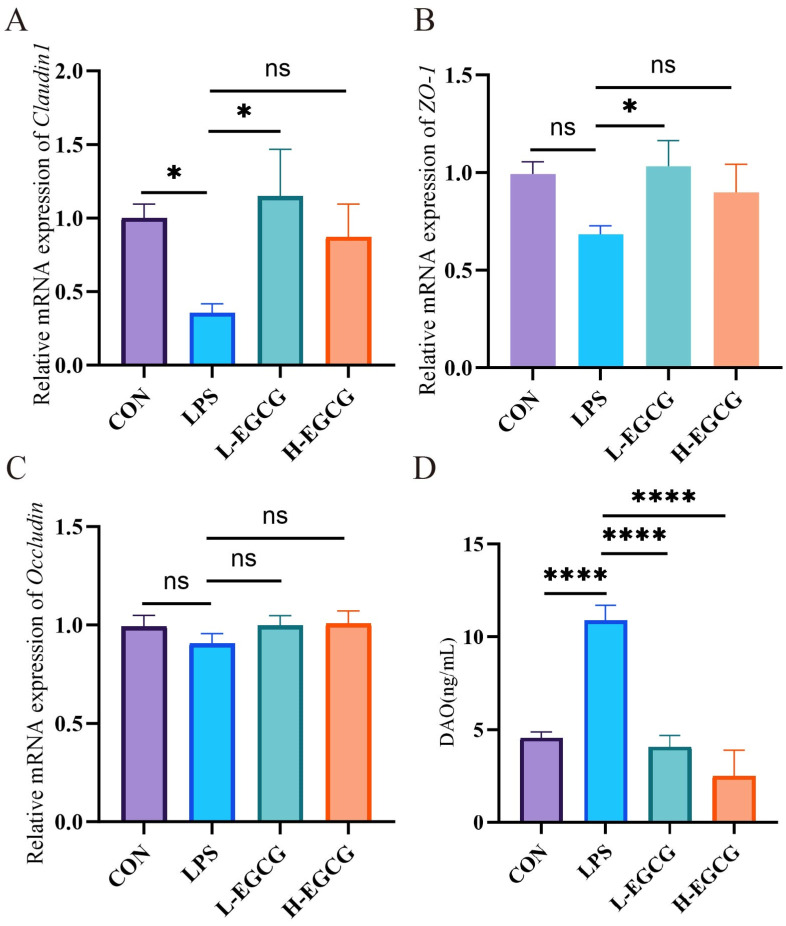
The effects of EGCG on the jejunum permeability of LPS-challenged Wenchang chicks. The mRNA expression levels of (**A**) *Claudin1*, (**B**) *ZO-1,* and (**C**) *Occludin* in the jejunum; (**D**) the serum diamine oxidase (DAO) concentration. The results are expressed as mean ± the standard error of the mean (* *p* < 0.05, **** *p* < 0.0001, not significant (ns) *p* > 0.05.).

Compared to controls, jejunal *Claudin1* expression was lower (*p* = 0.038, Figure 3A) and serum DAO content was higher (*p* < 0.001, Figure 3D) in the LPS treatment. The mRNA expression of *Claudin1* and *ZO-1* (*p* < 0.05, Figure 3A,B) were higher in the L-EGCG (40 mg/kg) treatment compared to LPS treatment. Moreover, serum DAO levels (*p* < 0.001, Figure 3D) were lower in the L-EGCG (40 mg/kg) treatment and H-EGCG (60 mg/kg) treatment.

### 3.4. Antioxidant Status of the Serum in LPS-Challenged Wenchang Chicks

The antioxidant results of the serum in LPS-challenged Wenchang chicks were shown in Table 3. CAT activity was lower (*p* < 0.001) in the LPS treatment compared to controls. Compared to LPS treatment, the T-AOC and CAT activities were higher (*p* < 0.05) and the MDA content was lower (*p* = 0.005) in L-EGCG (40 mg/kg) treatment, while the T-AOC and CAT activities were higher (*p* < 0.05) and the MDA content was lower (*p* = 0.014) in the H-EGCG (60 mg/kg) treatment.

### 3.5. Effects of EGCG on the Inflammatory Cytokine Levels in Serum and Jejunum of LPS-Challenged Wenchang Chicks

The changes in inflammatory cytokines in the serum and jejunum in LPS-challenged Wenchang chickens are shown in Table 4. In the LPS treatment, the IL-1β and TNF-α levels were higher (*p* < 0.001) while the IL-10 concentrations were lower (*p* < 0.001) in both the serum and jejunum compared to controls. IL-1β and TNF-α levels were lower (*p* < 0.001) and IL-10 concentrations were higher (*p* < 0.001) in the L-EGCG (40 mg/kg) treatment and H-EGCG (60 mg/kg) treatment compared to the LPS treatment.

### 3.6. Effects of EGCG on TLR4/MyD88/NF-κB Pathway Expression in LPS-Challenged Wenchang Chickens

**Figure 4:**
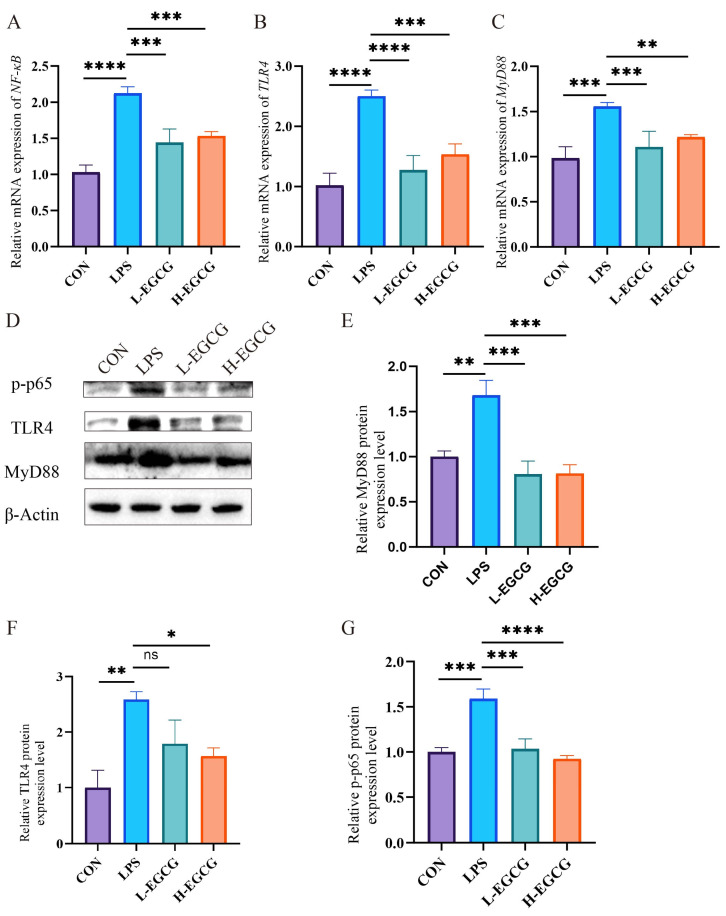
The effects of EGCG on the relative expression of *TLR4*/*MyD88*/*NF-κB* mRNA and protein levels in the jejunum of Wenchang chicks challenged with LPS. (**A**–**C**) The relative mRNA expression of *NF-κB*, *TLR4*, and *MyD88*. (**D**) Representative Western blot images. (**E**–**G**) The protein levels of MyD88, TLR4, and p-p65. The results are expressed as mean ± the standard error of the mean. (* *p* < 0.05, ** *p* < 0.01; *** *p* < 0.001, **** *p* < 0.0001, not significant (ns) *p* > 0.05). The original images of the Western blot are published as supplementary material.

In the LPS treatment, the jejunal *TLR4*, *MyD88*, and *NF-κB* mRNA expressions were higher (*p* < 0.01, Figure 4A–C) compared to controls. The mRNA expressions of these genes were lower (*p* < 0.05, Figure 4A–C) after EGCG supplementation.

A Western blot analysis showed that the jejunal TLR4, MyD88, and p-p65 levels were higher (*p* < 0.01, Figure 4E–G) in the LPS treatment compared to controls. MyD88 and p-p65 protein levels were lower (*p* = 0.001, Figure 4E,G) in the L-EGCG (40 mg/kg) treatment, while TLR4, MyD88, and p-p65 protein expression were lower (*p* < 0.05, Figure 4E–G) in the H-EGCG (60 mg/kg) treatment.

## 4. Discussion

Intestinal inflammation adversely affects poultry health and growth performance, resulting in significant economic losses [18]. Recent studies have demonstrated that dietary 300 mg/kg and 600 mg/kg EGCG supplementation alleviates heat stress-induced intestinal inflammation [13]. However, there were no reports confirming the efficacy of EGCG in ameliorating LPS-induced intestinal inflammation in Wenchang chickens, despite LPS being a major trigger of intestinal inflammation in poultry. Based on this knowledge gap, we established an LPS-induced intestinal inflammation model in Wenchang chickens to investigate EGCG’s regulatory effects on inflammatory response and the underlying mechanisms. This study provides novel insights into EGCG’s therapeutic potential against intestinal inflammation in Wenchang chickens.

LPS is a type of endotoxin that originates from Gram-negative bacteria. It has the ability to stimulate the innate immune system [19]. The intraperitoneal administration of LPS has been extensively documented as a well-established model for studying intestinal inflammation in animals. Multiple studies have constructed models of intestinal inflammation in broilers by intraperitoneal injection of 1 mg/kg BW LPS [14,20]. LPS-induced intestinal inflammation increases intestinal permeability and impairs barrier function, leading to reduced growth performance in broilers [14]. In our study, the LPS decreased growth performance, initially confirming the successful establishment of an intestinal inflammation model suitable for investigating EGCG effects on Wenchang chicken health.

EGCG is the main substance in green tea polyphenols. Studies have shown that dietary supplementation with 300 mL and 400 mL GTE or 300 mg/kg and 600 mg/kg EGCG can improve growth performance in broilers caused by coccidia infection and heat stress [12,21]. Our study, however, employed a different approach by administering EGCG orally rather than through feed supplementation. The oral administration method was selected based on multiple lines of evidence from previous research. Kiess et al. [22] demonstrated that oral delivery of bioactive compounds typically yields more pronounced physiological effects in broilers compared to conventional feed or water supplementation methods. Oral gavaging, feed addition, and water administration each have their own advantages and disadvantages. The benefit of oral gavaging lies in its ability to directly control the dosage, avoid intake variability among animals, and reduce metabolic losses in the oral cavity or esophagus. In contrast, the other two methods make it difficult to control the drug dosage for each experimental animal. Given the study’s focus on elucidating the molecular mechanisms by which EGCG prevents intestinal inflammation in Wenchang chickens, we selected oral gavaging to ensure precise dose delivery.

The specific dosage levels (40 and 60 mg/kg BW) were carefully chosen based on two key studies: Chi et al. [23] reported that supplementing drinking water with 40 mg/kg tea extract significantly enhanced both body weight gain and antioxidant capacity in broiler chickens, while Li et al. [24] found that oral administration of 60 mg/kg tea polyphenols led to significant increases in pancreatic lipase activity and reduced *IL-1β* expression. Building upon this foundational research, our study employed these evidence-based dosage levels to investigate EGCG’s biological effects and underlying mechanisms. Our findings align with previous research, demonstrating that oral EGCG administration significantly improves Wenchang chickens’ growth performance. However, further experimental studies through feed supplementation are still required to better guide the addition of EGCG in Wenchang chicken feed.

The development of the intestines has a substantial influence on the broilers’ ability to digest and absorb nutrients. Intestinal morphology, VH, CD, and VH:CD values are indicators of the structural integrity, developmental stage, and ability to absorb nutrients of the animal intestine [25]. An imbalanced intestinal barrier structure would result in the entry of bacteria, toxins, and other toxic substances into the body, leading to intestinal inflammation and impaired growth performance [26,27]. In our research, LPS-challenged broiler chicks had significantly reduced the jejunal VH, downregulated the expression of *Claudin1* and *ZO-1* mRNA in the jejunum and had a significant increase in serum DAO levels. Our findings align with previous research in this field. Notably, Zhang et al. [28] demonstrated that LPS exposure significantly downregulated the mRNA expression of genes encoding critical tight junction proteins, including *occludin*, *ZO-1*, *claudin-1*, and *claudin-2*. A dietary addition of GTE or EGCG can increase the mRNA expression levels of *Claudin1* and *Muc2* and decrease the VH and VH:CD in broilers induced by coccidial infection and heat stress [13,21]. In our research, we found that the gavaging of EGCG significantly increased jejunal VH, VH:CD, *Claudin1*, and *ZO-1* mRNA expression while also reducing the serum DAO levels. These results suggested that EGCG can improve the intestinal barrier, digestion, and absorption capacity of Wenchang chickens, thereby improving the growth performance of Wenchang chickens stimulated by LPS.

The health status of animals is highly correlated with their antioxidant capability [29]. When the body’s basic antioxidant defense system is compromised, oxidative stress damage occurs, potentially leading to intestinal diseases [30]. Our investigation discovered that intraperitoneal LPS administration significantly diminished antioxidant capacity, a finding corroborated by Lei et al. [31], who demonstrated that LPS exposure reduced antioxidant capacity and elevated MDA content. EGCG has exhibited significant antioxidant efficacy in numerous scientific investigations. Xue et al. [12] and Song et al. [13] independently reported that the dietary supplementation of 300 mg/kg and 600 mg/kg EGCG enhanced the antioxidant capacity in heat-stressed broilers. The antioxidant mechanism of EGCG is likely multifaceted. Hu et al. reported that green tea polyphenol extract and individual catechins can activate antioxidant-responsive element-dependent genes in transfected HepG2 cells [32]. Furthermore, Huang et al. demonstrated that EGCG exerts its antioxidant effects through activation of the Nrf2/HO-1 signaling pathway [33]. Our results were consistent with these findings, demonstrating that EGCG gavaging significantly increased T-AOC and CAT activities while decreasing MDA content in LPS-challenged Wenchang chickens. Moreover, after intragastric administration of EGCG, the activities of T-SOD and GSH-Px show an upward trend. These results indicate that EGCG effectively ameliorates LPS-induced antioxidant system imbalance, suggesting a potential mechanistic pathway through which EGCG mitigates LPS-induced intestinal inflammation in Wenchang chickens.

LPS-induced intestinal inflammation is characterized by the overproduction of inflammatory cytokines [34]. Our results showed that intraperitoneal injection of LPS significantly increased the levels of TNF-α and IL-1β in the serum and jejunum. The synthesis of these pro-inflammatory cytokines is typically initiated when LPS binds to TLR4 [35]. Specifically, TLR4 activation triggers the MyD88-dependent pathway, culminating in NF-κB activation and subsequent pro-inflammatory cytokine production [36]. In our experimental model, intraperitoneal LPS administration in Wenchang chicks led to significant upregulation of *TLR4*, *MyD88*, and *NF-κB* mRNA expression in jejunal tissue, accompanied by increased protein expression of TLR4, MyD88, and p-p65. These findings establish the TLR4/MyD88/NF-κB signaling cascade as a crucial mechanistic pathway in the LPS-induced intestinal inflammation model of Wenchang chicks. Based on these observations, we hypothesized that dietary interventions might ameliorate intestinal inflammation through modulation of the TLR4/MyD88/NF-κB pathway.

Indeed, our results confirmed that oral EGCG administration effectively suppressed both the mRNA and protein expression levels of *TLR4*, *MyD88*, and *NF-κB* components in jejunal tissue. This downregulation corresponded with reduced pro-inflammatory mediator levels in both serum and jejunal tissue, ultimately attenuating intestinal inflammation in chickens. In bovine ruminal epithelial cells (BRECs), EGCG pre-treatment significantly suppressed the LPS-induced upregulation of *TLR4* expression and activation of the downstream NF-κB pathways. A Western blot analysis demonstrated that EGCG directly reduced TLR4 protein levels and diminished its interaction with MyD88 [37]. In rat H9C2 cells, studies have shown that EGCG@Rh-gel effectively blocks the reactive oxygen species (ROS) inflammatory cycle through ROS scavenging and TLR4 inhibition [38]. Research has found that, in diabetic models, EGCG indirectly inhibits the stability of *TLR4* mRNA by promoting Fat Mass and Obesity-Associated Protein degradation, thereby reducing TLR4 protein expression [39]. These findings provide compelling evidence that EGCG mitigates LPS-induced intestinal inflammation in broilers primarily through downregulation of the TLR4/MyD88/NF-κB signaling pathway.

Furthermore, a comprehensive analysis of our experimental results demonstrated that both EGCG dosages (40 and 60 mg/kg BW) effectively improved growth performance, enhanced antioxidant capacity, and suppressed the TLR4/MyD88/NF-κB signaling pathway in LPS-challenged broilers. Since there were no significant differences between the two EGCG dosages in improving intestinal health and growth performance of Wenchang chickens, under these experimental conditions, we recommend the lower dosage of 40 mg/kg BW EGCG as an effective treatment for LPS-induced intestinal inflammation in Wenchang chicks.

Although this study confirmed the alleviating effects of EGCG on LPS-induced intestinal inflammation in Wenchang chickens, it should be noted that the oral gavage method used here (to explore underlying mechanisms) differs from practical production settings where EGCG is typically administered via feed or drinking water. Future studies should focus on comparing its optimal delivery routes and dose–response relationships under production conditions to facilitate its practical application in the poultry industry.

## 5. Conclusions

In conclusion, we demonstrated that EGCG alleviated LPS-induced intestinal inflammation and improved growth performance in Wenchang chicks. Furthermore, EGCG enhanced intestinal barrier functions and antioxidant capacities. The modulation of the TLR4/MyD88/NF-κB pathway by EGCG may play a critical role in its anti-inflammatory mechanisms. Our findings provide new insights into the ability of EGCG to promote intestinal health in Wenchang chicks and suggest that EGCG has the potential to serve as a dietary additive for enhancing intestinal health in Wenchang chickens.

## Figures and Tables

**Table 1:** Basal diet composition and nutrient level (%).

Ingredient	Content (%)
Corn	52.50
Soybean meal	40.00
Soybean oil	3.00
Dicalcium phosphate	1.90
Limestone powder	1.08
Salt	0.37
Lysine	0.05
Methionine	0.19
Premix ^(1)^	0.80
Choline chloride	0.11
Total	100.00
Nutritional levels ^(2)^	
Metabolizable energy (Kcal/kg)	2966.47
Crude protein	21.77
Calcium	1.0
Available phosphorus	0.44
Lysine	1.34
Methionine	0.55
Cystine	0.4

(1) Premix provided the following per kilogram of diet: VA 9000 IU; VD3 3000 IU; VE 26 IU; VK3: 1.20 mg; VB1 3.00 mg; VB2 8.00 mg; VB6 4.40 mg; VB12 0.012 mg; niacin 45 mg; calcium pantothenate 15 mg; folic acid 0.75 mg; biotin 0.20 mg; choline chloride 1100 mg; Fe 100 mg; Cu 10 mg; Zn 108 mg; Mn 120 mg; I 1.5 mg; and Se 0.35 mg. (2) CP was the measured value, while the others were all calculated values.

**Table 2:** List of genes and primer sequence for quantitative real-time PCR analysis.

Gene ^(1)^	Primer Sequences (5′ to 3′)	Length	Accession No.
*β-actin*	F-CAGCCAGCCATGGATGATGA	150	NM_205518.2
	R-CATACCAACCATCACACCCTGA		
*ZO-1*	F-CACTAGAGGATGAGGAGGAAGAAGAC	80	XM_040706827.2
	R-TACGCCACCATTGCTGTTGAATAC		
*Occludin*	F-CTTCGCCTGCGTCGCTTCC	104	NM_205128.1
	R-TGCCGTAGTAGTTGGAGCCGTAG		
*Claudin-1*	F-GGTATGGCAACAGAGTGGCT	91	NM_001013611.2
	R-CAGCCAATGAAGAGGGCTGA		
*NF-κB*	F-ACTTCTGGTGAAACACGGGG	80	NM_001001472.3
	R-CTCGTCCCCATGTAAGCTGG		
*MyD88*	F-AGCGTGCCAAAGACTTCAGA	201	NM_001030962.5
	R-ACACGTTCCTGGCAAGACAT		
*TLR4*	F-TGACCTACCCATCGGACACT	111	NM_001030693.2
	R-TGCCTGAGAGAGGTCAGGTT		

(1) ZO-1, zonula occludens-1; NF-κB, nuclear factor kappa-B; MyD88, myeloid differentiation primary response 88; TLR4, toll-like receptor 4.

**Table 3:** Impact of EGCG administration on antioxidant status in LPS-challenged Wenchang chickens.

Item ^(1)^	Treatments				SEM	*p*-Value ^(2)^
z	CON	LPS	L-EGCG	H-EGCG		
T-AOC (U/mL)	3.8 ^bc^	2.9 ^c^	9.9 ^a^	5.8 ^b^	0.67	<0.001
GSH-Px (U/mL)	1755	1677	1746	1834	25.10	0.172
T-SOD (U/mL)	388	370	412	421	8.42	0.124
CAT (U/mL)	32.7 ^a^	7.6 ^c^	15.3 ^b^	14.8 ^b^	1.98	<0.001
MDA (nmol/mL)	3.6 ^ab^	5.4 ^a^	2.2 ^b^	2.6 ^b^	0.40	0.027

(1) T-AOC, total antioxidant capacity; GSH-Px, glutathione peroxidase; T-SOD, total superoxide dismutase; CAT, catalase; MDA, malondialdehyde; SEM, standard error of the mean. (2) a,b,c means without common letters within the same row differ at *p* < 0.05.

**Table 4:** The effects of EGCG on cytokines content in the jejunum and serum of Wenchang chicks challenged with LPS.

Item ^(1)^	Treatments				SEM	*p*-Value ^(2)^
z	CON	LPS	L-EGCG	H-EGCG		
Serum						
IL-1β (pg/mL)	68.7 ^c^	95.8 ^a^	79.7 ^b^	83.0 ^b^	2.31	<0.001
TNF-α (pg/mL)	10.9 ^c^	14.3 ^a^	12.0 ^bc^	12.7 ^b^	0.30	<0.001
IL-10 (pg/mL)	9.3 ^a^	6.2 ^c^	8.2 ^ab^	7.5 ^b^	0.27	<0.001
Jejunum						
IL-1β (pg/mg)	53.1 ^c^	90.0 ^a^	66.4 ^b^	76.2 ^b^	3.03	<0.001
TNF-α (pg/mg)	6.1 ^c^	11.0 ^a^	6.9 ^c^	8.8 ^b^	0.38	<0.001
IL-10 (pg/mg)	13.1 ^a^	8.4 ^c^	12.4 ^a^	9.9 ^b^	0.40	<0.001

(1) IL-1β, interleukin-1β; TNF-α, tumor necrosis factor α; IL-10, interleukin 10; SEM, standard error of the mean. (2) ^a,b,c^ means without common letters within the same row differ at *p* < 0.05.

## Data Availability

All data are contained within the article.

## Supplementary Materials

The following are available online at https://www.mdpi.com/article/10.3390/vetsci12030225/s1, Figure S1: Measurement of the protein gray value.
