# Supplementary figures and images for: Epigallocatechin Gallate Alleviates Lipopolysaccharide-Induced Intestinal Inflammation in Wenchang Chicken by Inhibiting the TLR4/MyD88/NF-κB Signaling Pathway
Source: Vet Sci. 2025 Mar 2;12(3):225. doi: 10.3390/vetsci12030225 (PMC11945909; doi:10.3390/vetsci12030225)

Figure S1: Measurement of the protein gray value

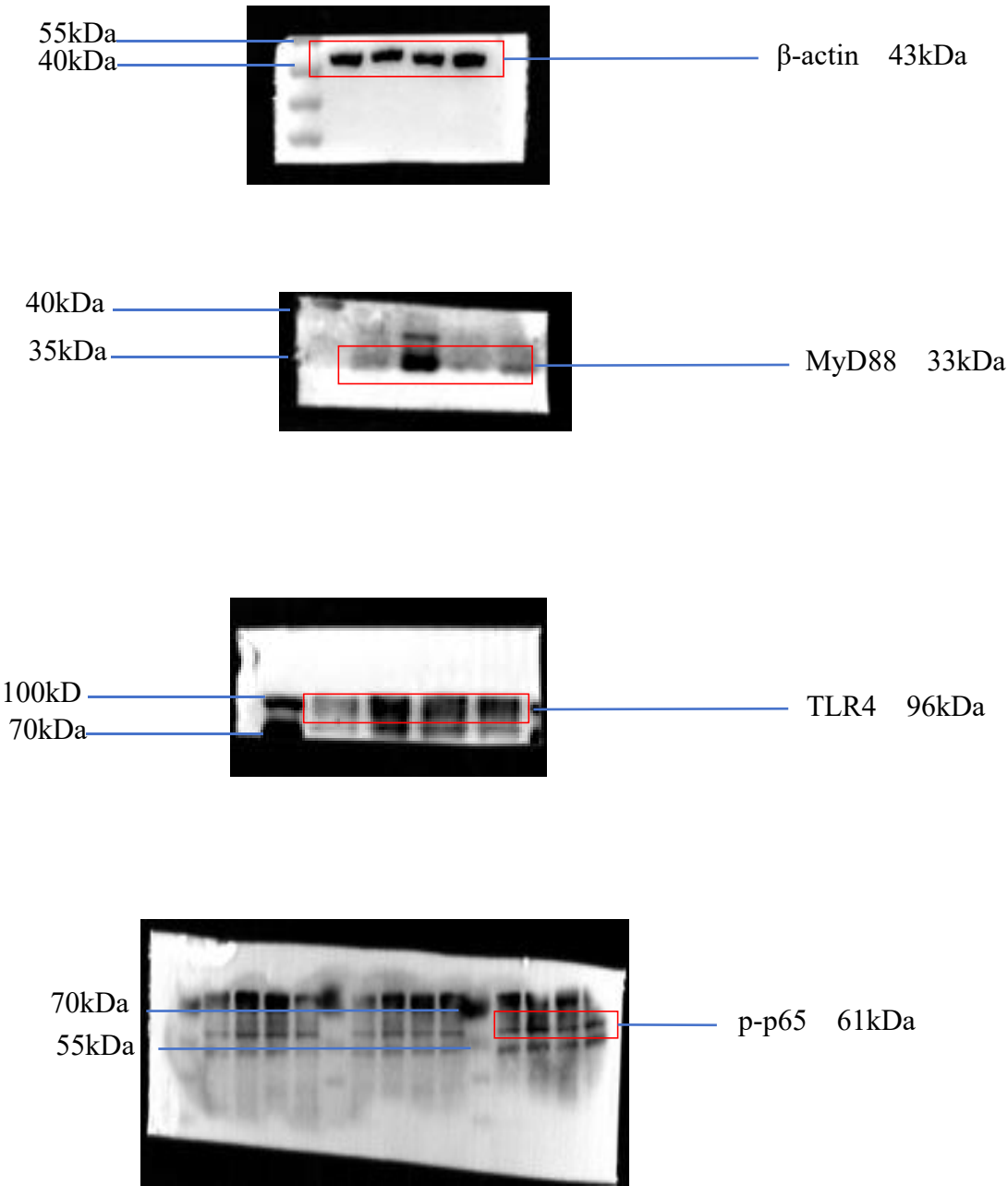

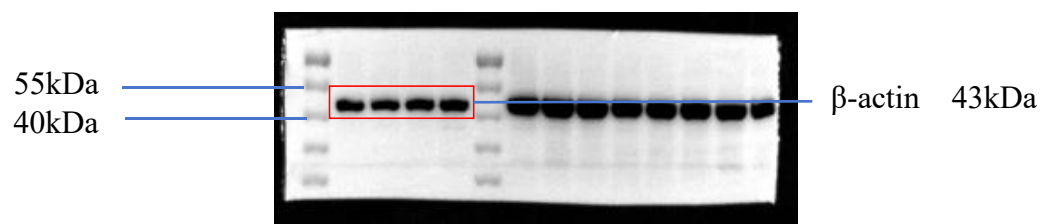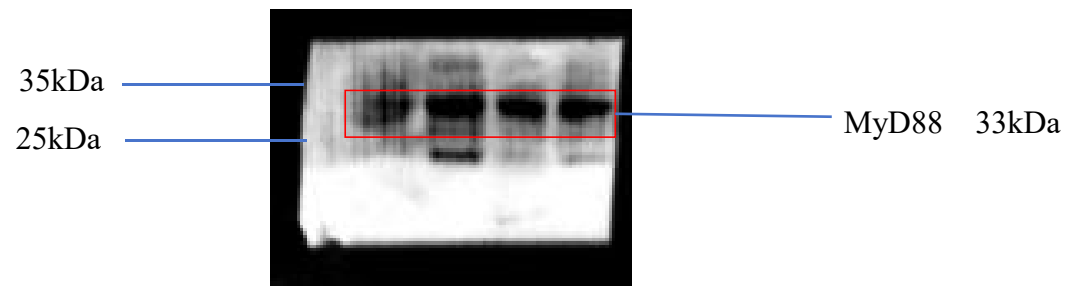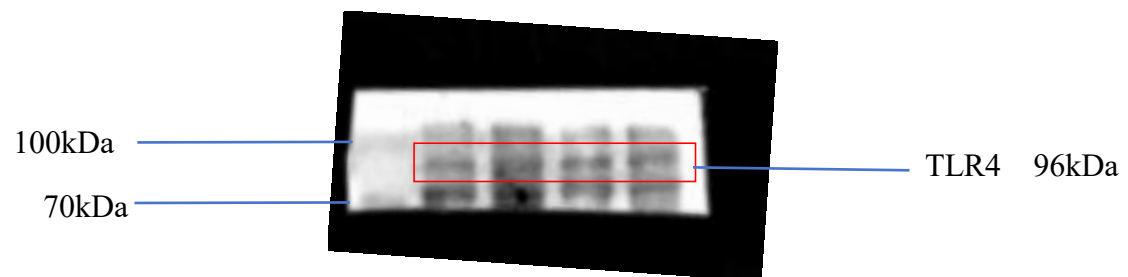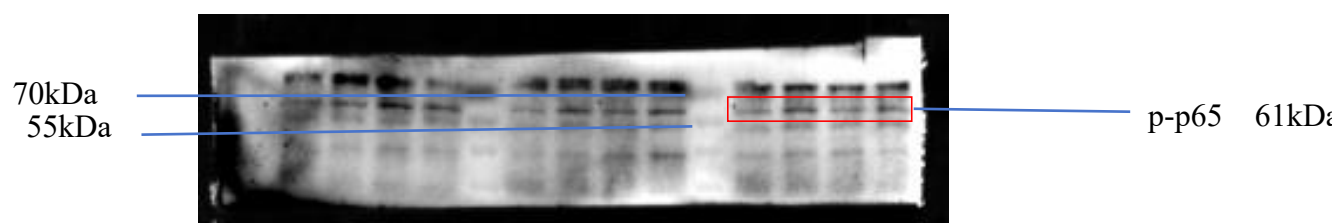

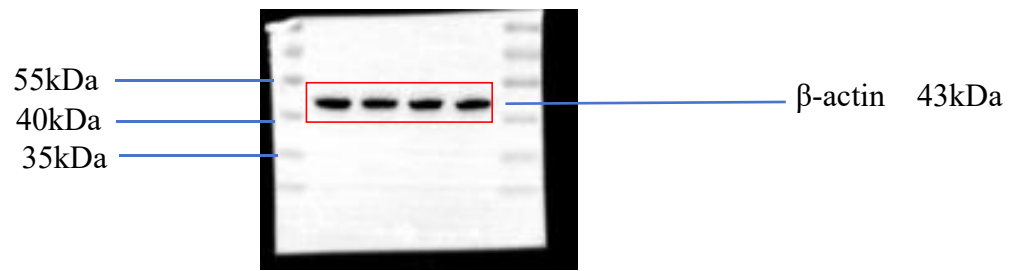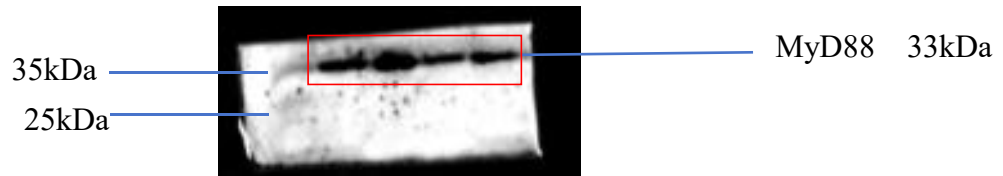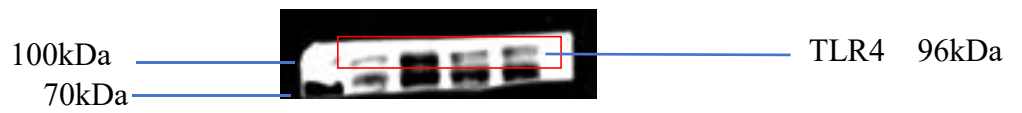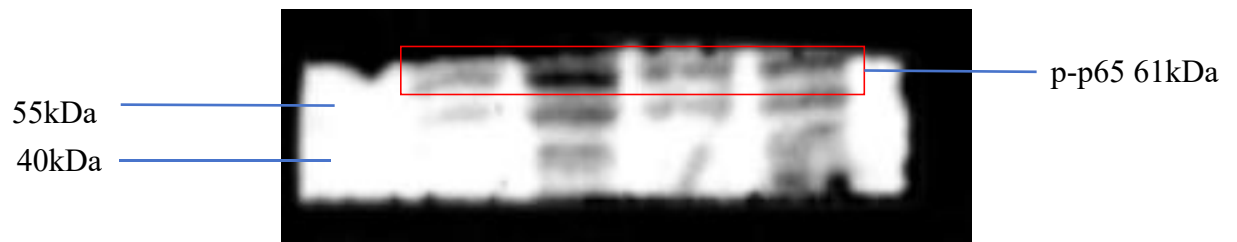

Supplement: Supplementary file 1 [file vetsci-12-00225-s001.zip › vetsci-3446257-supplementary.pdf]
